# Sulphur for Medicinal Uses

**Published:** 1888-11

**Authors:** 


### SULPHUR FOR MEDICINAL USES.

Sulphur fed in small quantities has been highly recommended as a preventive of abortion. Some farmers practice mixing a small proportion of sulphur with salt, which is kept in troughs where cattle, sheep and horses can have access to it at all seasons of the year. One pint of sulphur to four pints of salt is about the proportions recommended, although some prefer only about half that quantity of sulphur. A correspondent of an exchange, who has tested its merits, states that "for the last five years I have fed my cows and heifers sulphur in small quantities and at regular times, and I have never had a case of abortion in my herd, with the exception of one, and that was a cow I bought and brought into my herd in that condition. I expected trouble when I found out her condition, but there was not a single cow or heifer that was affected. Whether it is from feeding sulphur or not I will not say, but this I do say, while I have not had a case of abortion in my herd, my neighbors have all been bothered with this nuisance more or less." It is a simple, inexpensive remedy, and when not fed to excess can do no harm.

